# Relationship between Depressive Symptoms and Weather Conditions

**DOI:** 10.3390/ijerph19095069

**Published:** 2022-04-21

**Authors:** Agnė Brazienė, Jonė Venclovienė, Vidmantas Vaičiulis, Dalia Lukšienė, Abdonas Tamošiūnas, Irena Milvidaitė, Ričardas Radišauskas, Martin Bobak

**Affiliations:** 1 Institute of Cardiology, Lithuanian University of Health Sciences, Sukileliu Ave. 15, 50103 Kaunas, Lithuania; agne.braziene@lsmu.lt (A.B.); jone.vencloviene@lsmuni.lt (J.V.); dalia.luksiene@lsmuni.lt (D.L.); abdonas.tamosiunas@lsmuni.lt (A.T.); irena.milvidaite@lsmu.lt (I.M.); ricardas.radisauskas@lsmuni.lt (R.R.); 2 Department of Environmental Sciences, Vytautas Magnus University, Donelaičio St. 58, 44248 Kaunas, Lithuania; 3 Department of Environmental and Occupational Medicine, Lithuanian University of Health Sciences, Tilzes St. 18, 47181 Kaunas, Lithuania; 4 Health Research Institute, Lithuanian University of Health Sciences, Tilzes St. 18, 47181 Kaunas, Lithuania; 5 Department of Preventive Medicine, Lithuanian University of Health Sciences, Tilzes St. 18, 47181 Kaunas, Lithuania; 6 Institute of Epidemiology and Health Care, University College London, 1-19 Torrington Place, London WC1E 7HB, UK; m.bobak@ucl.ac.uk

**Keywords:** depressive symptoms, weather conditions, air temperature, wind speed, atmospheric pressure, relative humidity

## Abstract

Background: Weather is a well-known factor worldwide in psychiatric problems such as depression, with the elderly and females being particularly susceptible. The aim of this study was to detect associations between the risk of depressive symptoms (DS) and weather variables. Methods: 6937 participants were assessed in the baseline survey of the Health Alcohol Psychosocial Factors in Eastern Europe (HAPIEE) study during 2006–2008. To assess the risk of DS, a multivariate logistic model was created with predictors such as socio-demographic factors, health behaviors, and weather variables. Results: DS were found in 23.4% of the respondents, in 15.6% of males and in 29.9% in females. A higher risk of DS (by 25%) was associated with November–December, a rising wind speed, and relative humidity (RH) < 94% and snowfall during the cold period occurring 2 days before the survey. A higher air temperature (>14.2 °C) predominant during May–September had a protective impact. A higher risk of DS in males was associated with lower atmospheric pressure (<1009 hPa) 2 days before. Females were more sensitive to the monthly variation, snowfall, and RH. Conclusions: The findings of our study suggest that some levels of weather variables have a statistically significant effect on DS.

## 1. Introduction

A number of health disorders such as cardiovascular [1,2], respiratory [3,4], or nervous system diseases [5,6,7] are related to weather conditions. Meteorological factors affect both physical and mental health [8,9]. One of the most relevant psychiatric disorders today is depression [10]. The development of depression is affected by many environmental factors, such as increased ambient air pollution [11,12,13], geomagnetic storms [14], seasonal variation [15], and traffic noise [16].

Depression is a highly prevalent risk factor for cardiovascular diseases and is associated with morbidity and mortality from these diseases [17,18,19]. The diagnosis of depression and higher levels of depressive symptoms predicted elevated mortality in cancer patients [20], was associated with the development of dementia [21] and hospital readmission in older adults with asthma and chronic obstructive pulmonary disease [22], aggravated the prognosis of Parkinson’s disease [23], and negatively influenced quality of life [24,25].

One of the environmental factors that constantly draw the attention of researchers is how seasonal variation and weather changes affect mood and depressive symptoms (DS) [26], yet these results are inconsistent in different studies. Some found elevated DS in winter [15,27,28], while the effect of seasonality on DS was not observed in other large-scale studies [29], or the effect of seasonality was dependent on sex [30,31]. In addition, depressive symptoms are common and insufficiently investigated in older adults. Among these, mood may be particularly vulnerable to the effects of changing environmental stimuli owing to lifestyle considerations (e.g., reduced physical activity in winter months) but also due to changing neurophysiology that accompanies aging (e.g., altered circadian rhythms). Much work has investigating seasonality in the epidemiological literature, but has not extended to include older age groups or has focused on particular age extremes [15]. According to a large review study performed by Italian researchers, this delay in studies on climate change and their mental health consequences is significant. The lack of literature is perhaps due to the complexity and novelty of this issue [32]. The aim of this study was to detect the associations between the risk of DS and weather variables. It was conducted to answer the following questions: (a) do weather variables increase the risk of DS; (b) is the association between weather variables and depressive symptoms stronger in women than in men; (c) is the association between weather variables and depressive symptoms stronger in the elderly than in young adults?

## 2. Materials and Methods

### 2.1. Study Description

The study was performed in Kaunas, the second largest city of Lithuania. This article presents data from the framework of the international Health, Alcohol and Psychosocial Factors in Eastern Europe (HAPIEE) study conducted during 2006–2008. The participants were randomly selected by sex and age (45–74 years) from the Kaunas population register database. In total, 7077 respondents participated in this survey. The final study sample consisted of 6937 participants, because in 140 (2%) cases no data on DS were available. Information on other variables was obtained by using a structured questionnaire with questions about education, marital and smoking status, alcohol consumption, physical activity, antihypertensive treatment, and other factors. DS was evaluated using the 10-item Center for Epidemiologic Studies Depression Scale, (CES-D 10) [33,34]. All the participants were asked to answer 10 questions to evaluate the presence of DS during the past week. Each answer was rated as either 0 or 1, 0 meaning “no”, and 1 meaning “yes”. The maximal possible sum of the evaluation points ranged from 0 to 10. If the sum of the scores was equal to or greater than 4, patients were regarded as having DS [35]. The survey was performed during January–June and during September–December.

During the studied period, the main Kaunas meteorological monitoring station (Air Force Datsav3 station number: 266290; DMS coordinates 54°53′02.7″ N 23°50′09.2″ E) (hereafter—Station No. 1) located 6 km to the west of Kaunas city center provided daily records of minimum, maximum, and mean daily air temperature (T, °C), dew point temperature (°C), wind speed (WS, knots (kt)), atmospheric pressure (AP, hPa), and snowfall days. The apparent temperature (AT), defined as a person’s perceived air temperature, was calculated from the air and dewpoint temperature [36] by using the following formula: −2.653 + 0.994 × (air temperature (°C)) + 0.0153 × (dew point temperature (°C))^2^. The information on mean daily relative humidity (RH, %) was obtained from Kaunas International Airport weather station (Air Force Datsav3 station number: 266295; DMS coordinates 54°57′50.4″ N 24°05′06.0″ E) (hereafter—Station No. 2) located 14 km northeast of Kaunas city center. Both of the aforementioned weather stations are located on the outskirts of Kaunas city.

### 2.2. Statistical Analysis

First, we detected clinical and behavioral variables that were significantly associated with DS. To assess the crude association between sociodemographic, health behavior, and categorical weather variables and the prevalence of DS, the χ^2^ test was used. Second, we analyzed the crude associations between the weather pattern and the risk of DS. As the dependent variable DS is binary (yes, no), to assesses the crude association between continuous weather variables and the prevalence of DS, univariate logistic regression was used. In the analysis, as predictors, we used the mean values of weather variables and their range (maximal value-minimal value) on the day of the survey and on 2 previous days (with a lag of 0–2 days). The terms for weather variables were used as continuous or categorized. The cut-offs of categorical variables were detected by using the classification and regression tree (CRT, http://links.lww.com/HJH/B6 (accessed on January 2022)) method [37]. The cut-offs for air temperature variables were detected by the first split of notes, and for other weather variables by 1–2 splits. We investigated the effect of weather variables throughout the study period and separately during the cold (November–March) and warm (April–October) periods. To assess the risk of DS, a multivariate logistic model was created with predictors such as statistically significant demographic factors, health behavior, and categorical weather variables:

$$P\{Y=1|X=x\}=\frac{\exp(g(x))}{1+\exp(g(x))},\;\mathrm{where}\;g(x)=\beta_{0}+\beta_{1}x^{\left(1\right)}+\beta_{2}x^{\left(2\right)}+\ldots +\beta_{k}x^{\left(k\right)},$$

where *P*{Y = 1|X *=* x} is the probability of the presence of a DS, x^(i)^, I = 1, 2,…, k are the socio-demographic, health behavior, and weather variables, β_i_ are the regression coefficients, and (exp(β_i_) is adjusted OR). The final model for DS included only weather variables whose *p*-value of the adjusted odds ratio (OR) was less than 0.05. The effect of the weather variables was also assessed separately for males and females.

The statistical analysis was performed using SPSS 19.0 software. The indicators of risk are presented as odds ratios (OR) with 95% confidence intervals (CI) and the *p*-value.

## 3. Results

We investigated the data of 6937 participants, 3145 males (45.3%) and 3792 females (54.7%), whose age ranged from 45 to 74 years (mean age, 58.8 years, SD = 7.5 years). DS were found in 1623 (23.4%) of the respondents. In males, DS were observed in 15.6% of the respondents, but in females, DS occurred almost twice as often, i.e., in 29.9% of the participants. Table 1 presents demographic, social, and other risk factors for the presence of DS. Almost half of participants (45.7%) were interviewed during the cold period. The rate of DS during cold and warm periods was similar, 23.5% and 23.3%, respectively.

During 2006–2008, the median (the first and the third quartiles) for T was 7.3 (2.9; 12.4) (°C), for AP 1008 (1015; 1021) (hPa), for RH 83 (71; 91) (%), and for WS 6.9 (5.0; 8.7) (kt). A higher rate of DS was observed during March–April (25.1%) and during November–December (25.2%) as compared to other months (22.3%) (*p* = 0.024).

**Figure 1:**
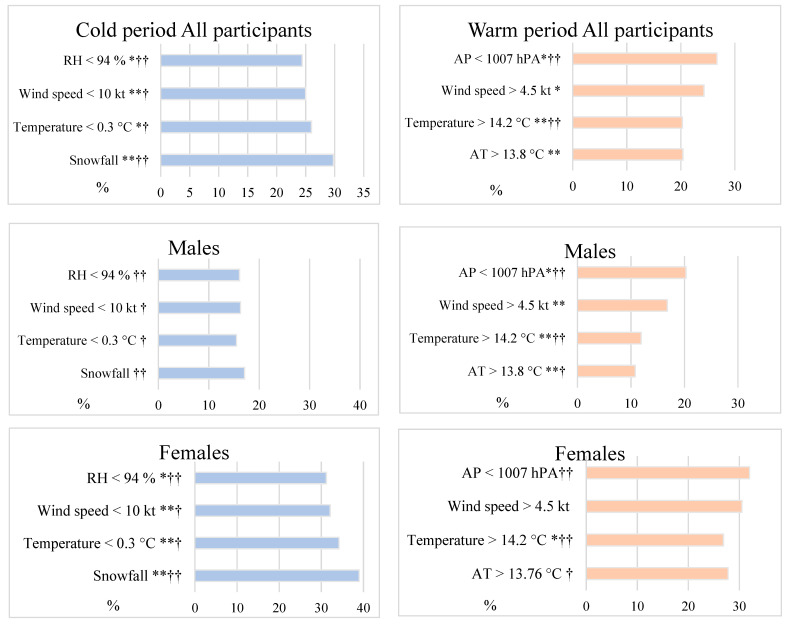
The distribution of the rate of DS by meteorological factors during the cold and warm periods. * *p* < 0.05, ** *p* < 0.01; †—the day before the survey; ††—2 days before the manifestation of depressive symptoms; RH—relative humidity; AP—atmospheric pressure; AT—apparent temperature.

During the cold period, snowfall 2 days before the survey had the greatest impact on the risk of DS (29.8%). A similar influence on the risk of DS was found when the mean day air temperature on the previous day was lower than 0.3 °C (26.0%), RH was lower than 94% with a lag of 2 days, and wind speed was lower than 10 m/s per day before the survey (Figure 1). A negative association between WS with a lag of 1 day and the risk of DS was found (crude OR = 0.97 95% CI 0.91–0.99). However, the effect of these weather variables was statistically significant only in females (Figure 1).

Mostly during the warm period, DS was observed when AP two days before the survey was lower than 1007 hPA (26.7%) and when the WS was higher than 4.5 m/s (24.3%) (Figure 1). A statistically significant effect of higher WS and lower AP and AT was found only in males.

**Figure 2:**
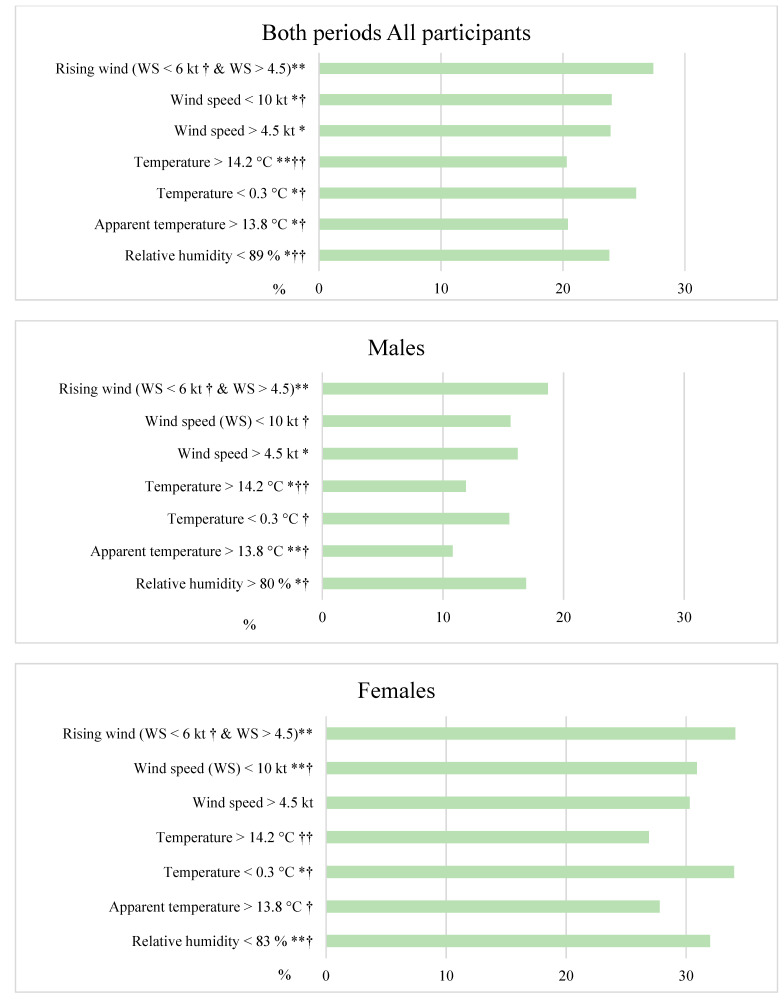
The distribution of the rate of DS by the meteorological factors during the entire period. * *p* < 0.05, ** *p* < 0.01; †—the day before the manifestation of depressive symptoms; ††—2 days before the manifestation of depressive symptoms; WS—wind speed.

Throughout the studied period, rising wind, if WS on the previous days was lower than 6 kt (40th percentile) (27.4%) (Figure 2), had the greatest influence on the risk of DS, both for males and females.

A similar effect on the manifestation of DS was observed when WS was higher than 4.5 m/s (23.9%) and WS was lower than 10 m/s (24%), but for males and females WS variables had different effects. In females, a higher rate of DS was observed if RH on the previous day was lower than 83%, while in males, such effect was observed when RH on the previous day was higher than 80% (Figure 2). During both periods, DS was the rarest when ambient temperature was higher than 14.2 °C (20.3%) two days before the survey (Figure 2). A higher T had a stronger effect in males, but a T lower than 0.3 °C had a significant effect in females. For males, AP lower than 1009 hPa two days before the survey was associated with a higher rate of DS (17.8%).

According to the multivariate logistic model for all the participants, the risk of DS was related to female sex (OR = 2.19, 95% CI 1.90–2.54), primary or vocational education (OR = 1.57, 95% CI 1.31–1.88), and physical inactivity (OR = 1.49, 95% CI 1.31–1.70). During November–December, the risk of DS increased by 1.25 times. ORs of DS were related to the RH lower than 93.5% (OR = 1.32, 95% CI 1.07–1.61) and to the rising wind if WS on the previous days was lower than 6 kt (OR = 1.30, 95% CI 1.14–1.49) (Table 2). Snowfall during the cold period increased the odds ratio (OR) of DS manifestation by 1.28 times (95% CI 1.01–1.64).

## 4. Discussion

According to our findings, a higher risk of DS was associated with November–December and a weather pattern related to a colder period such as snowfall, especially for females. Apart from this, a higher T (>14.2 °C) that was predominant during May–September had a protective impact. The effect of seasonality was stronger in females. These results corroborate those obtained by other authors. The study with a repeated measurement design [31] showed a higher depression score measured by the BDI instrument during the non-summer period, and the effect of seasonality was stronger in females.

We found that a higher rate of DS was observed on “rising WS” days, if WS on the previous days was lower than the 40th percentile” and, for females, on days after a lower RH. During the study period, these weather patterns were more common during the non-winter period, especially during summer, and were associated with a lower RH and a lower cloud level. Therefore, these conditions may be associated with a lower level of negative air ions during the non-winter period. The results of the meta-analysis showed that a higher level of negative air ions (negative air ionization) was associated with lower depression scores [38]. During the days with higher RH, there was possibly more precipitation, and, at the same time, negative air ion concentration increased [39]. Apart from this, days of RH > 93% during the study period were associated with a lower daily AP, a higher rate of precipitation, and with higher cyclonic activity. It is probable that these days may be associated with an increase in negative air ion (NAI) exposure. NAIs were generated from the surrounding air molecules by charging themselves negatively when water droplets collide with each other or with a wetted solid to form a fine spray of drops [40]. A higher negative air ion concentration during wetter days due to the amount of precipitation (Lenard effect) [40] on the previous day may be linked to a lower score for DS in females. Evidence has shown that NAIs could significantly reduce the level of serotonin in blood or brain [40,41]. The results of other studies also showed a positive effect of rain on mental health. Rainfall decreased the risk of panic anxiety disorders [9]. Anxiety episodes seemed to increase just before a storm set in and to decrease after the onset [42].

According to the multivariate model, males were more sensitive to changes in AP, whereas females were more sensitive to seasonal variation, snowfall, and RH. For males, vice versa, a higher RH was associated with an elevated rate of DS, but this effect was non-significant in the multivariate model. Meanwhile, a higher rate of DS was observed in males two days after a lower AP. These weather conditions are usually associated with cyclonic conditions and rain and snow, which may change human activities and cause stress and DS, especially in males. Research conducted by other authors showed that low AP was associated with an increase in impulsive behaviors [43], migraine attacks in patients with migraine [44], and suicides [45]. Apart from this, males were found to be more prone to attempt suicide under low AP, while females exhibited such a trend under high AP [46]. In addition, animal experimental models showed that lowering barometric pressure aggravated depression-related behaviors [47]. These results are in line with our findings.

We found that a higher rate of DS was observed on “rising WS” days if WS on the previous days was lower than the 40th percentile”. During the warmer period, this pattern was associated with a higher daily mean T and WS on the days of the survey and a higher fall in the daily AP. These conditions were associated with warmer and dry winds that are known to increase the concentration of positive ions in the atmosphere [9], which increases blood and brain serotonin levels [48]. During the colder period, these conditions of rising WS were associated with a lower T, a higher AP, and higher changes in the daily AP, i.e., with colder air. The elevation of positive air ions during the warmer period and with colder air might be relevant factors in the correlation between these changes in WS and DS.

A number of studies found differences between men and women in health effects and mortality from cardiovascular diseases in relation to weather conditions and other meteorological factors [49,50,51]. The strong male/female difference in depression is well-known and is often reported in the literature [52,53,54]. One study in the south of the Netherlands showed that men had seasonal peaks of major depression and sad mood in the summer, while women had seasonal peaks in the fall [55]. This is in contrast to our study, where a higher rate of DS in females was observed during March–April. Many theories suggest that the variations in serotonin level could lead to the development of many neurological and psychiatric disorders, including depression [56]. Patients with depression are known to show a significant reduction in the levels of 5-hydroxyindoleacetic acid (5-HIAA), 5-HIAA being a serotonin metabolite produced in the brain from tryptophan, which is among the few amino acids that reach the central nervous system [56]. Circannual variation in 5-HIAA fitted with a spring-peak; according to the non-parametric regression, especially during March–April and with a minimum in summer [57]. The correlation between 5-HIAA seasonality and DS was found to be statistically significant [57]. It is probable that a higher rate of DS in females during March–April was due to annual fluctuations in the serotonin level.

Several recent studies have suggested promising ideas for future work. One of these is a cross-sectional study in Poland which revealed that medium positive correlations were found between meteoro-sensitivity/meteoropathy and cyclothymic and anxious temperaments. Small positive correlations were revealed between depressive and irritable temperaments and both meteoro-sensitivity and meteoropathy scales. No correlation was found between hyper-thymic temperament and meteoro-sensitivity/meteoropathy. The results also suggest that affective temperaments may be more related to meteoropathy symptoms in women [58], and also with stress [59]. Another new study, performed as a cross-over study, evaluated the effect of acute stress induced with the socially evaluated cold pressor test (SECPT) on cortical meta-plasticity in humans using a non-invasive brain stimulation protocol. The results showed that SECPT induced cardiovascular adaptations (increase in systolic, diastolic blood pressure, and heart rate), indicating that SECPT effectively induced acute stress. These experiments’ stimulation of subjects with sub-threshold 1-Hz repetitive stimulation (rTMS) after they had undergone the SECPT condition also induced inhibition of evoked potential (MEP), whereas 1-Hz rTMS administered after the control condition induced a facilitatory (physiologic) response pattern. Here we observed that acute stress impairs homeostatic meta-plasticity. In men, the dysfunctional regulation of cortical plastic changes after stress could play a pivotal role in the pathogenesis of neurological and psychiatric diseases [60].

Meanwhile, we found a statistically significant effect of some levels of weather variables on DS, but the effect of continuous weather variables was non-significant. Other researchers did not find any statistically significant meteorological predictors for DS [28], possibly because they used quantitative meteorological variables in the regression models, or longer-term averages were used that did not reflect short-term changes in meteorological conditions. Since the effects of potentially non-linear meteorological variables are observed, the use of categorical predictors gives better results.

## 5. Limitations

There are some limitations to our study. One is that the survey was not performed during July-August—the hottest summer months, and therefore the effect of heat was not evaluated. Another limitation to the presented study is the cross-sectional nature of the design. In common with the majority of epidemiological studies, we did not apply diagnostic interviews for depression but used a self-report questionnaire for the evaluation of the frequency of DS. We did not have the opportunity to obtain all the meteorological data we need from one meteorological station, or from both stations. Therefore, we used different meteorological indicators from the separate meteorological stations (from Station No. 1—daily air temperature, dew point temperature, wind speed, atmospheric pressure, and snowfall days; from Station No. 2—relative humidity). However, given the quite small size of the territory of the Kaunas region and the even population distribution, this is unlikely no influence our results. Using the results of a single series of weather conditions for the entire city can lead to exposure misclassification. One more limitation is that we did not analyze the role of other lifestyle factors such as the frequency of alcohol use, stress and burnout rates at work and at home, unemployment, working conditions, or antidepressant use, which could influence the outcome of DS. Finally, this study represents only one city and urban population, and thus the results cannot be reasonably generalized to the whole population of Lithuania.

## 6. Conclusions

The results of our study showed a statistically significant effect of some levels of weather variables (daily air temperature, wind speed, atmospheric pressure, and relative humidity) on depressive symptoms, but the effect of continuous weather variables was non-significant.

## Figures and Tables

**Table 1:** Distribution of participants with depressive symptoms according to risk factors.

Characteristic		Depressive Symptoms *N* (%)		*p* Value
		No	Yes	
Sex	Male	2655 (84.4)	490 (15.6)	<0.001
	Female	2659 (70.1)	1133 (29.9)	
Age groups (years)	<50	635 (79.1)	168 (20.9)	0.037
	50–69	3994 (76.7)	1213 (23.3)	
	≥70	685 (73.9)	242 (26.1)	
* BMI (kg/m²)	<25	1095 (76.9)	329 (23.1)	<0.001
	25–29.9	2153 (78.8)	579 (21.2)	
	≥30	2066 (74.3)	715 (25.7)	
Smoking status (male)	Never	1052 (86.7)	163 (13.3)	0.012
	Former	813 (83.7)	158 (16.3)	
	Regular/occasional	790 (82.2)	171 (17.8)	
Smoking status (female)	Never	2221 (70.5)	931 (29.5)	0.533
	Former	183 (67.5)	88 (32.5)	
	Regular/occasional	254 (69.0)	114 (31.0)	
Marital status	Single	200 (71.9)	78 (28.1)	<0.001
	Married	3889 (81.6)	878 (18.4)	
	Divorced	565 (67.0)	278 (33.0)	
	Widowed	597 (61.8)	369 (38.2)	
	Cohabiting without marriage	63 (75.9)	20 (24.1)	
Education	Primary	350 (71.0)	143 (29.0)	<0.001
	Vocational	469 (72.2)	181 (27.8)	
	Secondary	1454 (75.4)	475 (24.6)	
	Advanced vocational	1193 (74.9)	400 (25.1)	
	University	1848 (81.3)	424 (18.7)	
Ischemic heart disease	Yes	935 (70.1)	399 (29.9)	<0.001
	No	4379 (78.2)	1224 (21.8)	
Medications taken for high blood pressure during the last 2 weeks	Yes	1993 (73.0)	739 (27.0)	<0.001
	No	3310 (79.0)	881 (21.0)	
Physical activity	Yes	4047 (78.1)	1136 (21.9)	<0.001
	No	1264 (72.3)	485 (27.7)	

* BMI—body mass index.

**Table 2:** The risk of depressive symptoms in association with some sociodemographic and lifestyle factors, illnesses, and meteorological factors, adjusting for age.

Variable	OR (95% CI)	*p*
Female sex	2.19 (1.90–2.54)	<0.001
Smoking (former/regular/occasional)	1.19 (1.03–1.37)	0.015
Cohabiting without marriage	1	
Single	1.40 (1.06–1.86)	0.020
Divorced/widowed	1.92 (1.69–2.18)	<0.001
Education (university)	1	
Education (primary/vocational)	1.57 (1.31–1.88)	<0.001
Education (secondary/advanced vocational)	1.36 (1.19–1.56)	<0.001
IHD	1.39 (1.21–1.61)	<0.001
AH	1.25 (1.11–1.41)	<0.001
Physical inactivity	1.49 (1.31–1.70)	<0.001
November-December	1.25 (1.06–1.48)	0.008
Relative humidity < 94% with a lag of 2 days	1.32 (1.07–1.61)	0.009
Rising wind (WS on the previous day < 6 & WS > 4.5)	1.31 (1.14–1.49)	<0.001
Snowfall during the cold period 2 days before	1.28 (1.01–1.64)	0.042
Temperature > 14.2 °C with a lag of 2 days	0.79 (0.67–0.93)	0.006

IHD—ischemic heart disease, AH—arterial hypertension. For males, rising WS and AP below 1009 hPa were associated with a higher risk of DS (ORs with 95% CI were 1.40 (1.11–1.75) and 1.28 (1.04–1.59), respectively), and a higher T had a protective effect (Table 3). For females, a higher risk of DS was observed during March–April and November–December, two days after the snowfall, a day after RH < 82.5%, and on days of some variation in WS described as rising WS (Table 3).

**Table 3:** The risk of depressive symptoms depending on meteorological factors according to sex (adjusting for age).

Variable	OR (95% CI)	*p*
Males		
Rising wind (WS on the previous day < 6 & WS > 4.5)	1.40 (1.11–1.75)	0.004
Temperature > 14.2 °C with a lag of 2 days	0.69 (0.51–0.93)	0.013
Atmospheric pressure < 1009 hPa with a lag of 2 days	1.28 (1.04–1.59)	0.023
Females		
March–April	1.24 (1.04–1.49)	0.020
November–December	1.38 (1.12–1.70)	0.002
Rising wind (WS on the previous day < 6 & WS > 4.5)	1.28 (1.09–1.51)	0.003
Snowfall at cold period before 2 days	1.55 (1.16–2.07)	0.004
Relative humidity < 83% on the previous day	1.26 (1.08–1.48)	0.004

## Data Availability

Data are not accessible online.
